# Supplementary material for: Selective Anticancer Therapy Based on a HA-CD44 Interaction Inhibitor Loaded on Polymeric Nanoparticles
Source: Pharmaceutics. 2022 Apr 4;14(4):788. doi: 10.3390/pharmaceutics14040788 (PMC9032636; doi:10.3390/pharmaceutics14040788)
Supplement: Supplementary file 1 [file pharmaceutics-14-00788-s001.zip › pharmaceutics-1647824 Supplementary Materials.pdf]

pharmaceutics

MDPI

Supplementary Materials: Selective Anticancer Therapy Based on a HA−CD44 Interaction Inhibitor Loaded on Polymeric Nanoparticles

Jose M. Espejo−Román, Belén Rubio−Ruiz, Victoria Cano−Cortés, Olga Cruz−López, Saúl Gonzalez−Resines, Carmen Domene, Ana Conejo−García and Rosario M. Sánchez−Martín

LIST OF CONTENTS

1. Computational studies S2

2. Synthesis and characterization S11

3. General experimental methods S14

3.1. Synthesis and characterization of **Naked−NPs** (**1**) S14

3.1.1. Characterization of **Naked−NPs** (**1**) S14

3.2. Determination of NPs concentration (NPs/μL) by spectrophotometric method S15

4. Summarized table of the synthesized nanoparticles S16

5. Characterization of **JE22−NPs** (**5**) before and after incubation in PBS pH= 5 for 5 days by DLS S16

6. Stability study of **JE22−NPs** (**5**) by zeta potential analysis S17

7. Absorbance and calibration standard curves of **JE22** of **JE22−NPs** (**5**) S18

8. Fluorescent labelling of **JE22−NPs** (**5**) S18

9. Flow cytometry analysis of CD44 expression in MDA−MB−231 and MCF−7 cells S19

10. Effect of **JE22−NPs** (**5**) on cell viability in MCF−7 cell line S19

11. Competitive assay of **JE22−NPs** (**5**) in MDA−MB−231 cell viability S20

12. Dose−response curve of MDA−MB−231 treated with **JE22−NPs** (**5**) S20

13. Study of pH effect in MDA−MB−231 cell viability S21

14. Cytotoxic analysis of **JE22−NPs** (**5**) in non−cancerous HEK−293 cell line S21

15. Confocal microscopy analysis of **JE22−NPs** (**5**) with CD44+cells S22

16. Effect of **JE22−NPs** (**5**) on apoptosis in MCF−7 cell line S22

17. References S23

1. Computational studies

The charges and parameters for the ligands were searched using the CHARMM−GUI ligand modeller interface that generates the ligand force field parameters and necessary structure files by searching for the for small molecules in the verified CHARMM force field library or using the CHARMM general force field (CGenFF). The results suggested the need to optimize the charges and certain dihedral angles as the penalties were high. The parameters and partial changes for **THIQ−ester** and **JE22** (THIQ−ketone) were optimized using the FFTK plugin of VMD as an input generator and a refinement tool. CGenFF 36 parameters used are reported in Tables S1 to S8, and atom types in Scheme S1. The equilibrium bonds and angles were obtained after geometry optimization at the MP2/6−31+G(d) level of theory. The target data for the charge validation/optimization was obtained from the optimization of the distances and interaction energies between water molecules and the ligand following the standard CHARMM protocol [1]. For the charge optimization, the aliphatic hydrogen atoms were constrained to a partial charge of 0.09 and the aromatic hydrogens of the benzene ring were constrained to 0.115. Bond, angles and improper force constants did not required optimization considering the penalties reported by Charmm−Gui in the 'Ligand reader and modeler for CHARMM Force Field generation of small molecules' module. QM interaction−energy calculations were performed at the MP2/6−31+G(d) level of theory, with diffuse basis functions on non−H atoms (C, O) to correctly estimate interaction energies for an overall system charge. The resulting QM potential energy surface (PES) was used as the reference benchmark, and CHARMM MM partial charges were fitted onto this QM PES with the MCSA algorithm. Torsional potential optimization was done by QM PES scans of magnitude χ = 90° in intervals of 15 degrees. The dihedrals optimized in the **THIQ−ester** ligand were [OG302−CG2O2−CG321−CG321], [CG321−CG2O2−OG302−CG331], [CG2R61−CG321−CG321−NG301], [CG2R61−CG321−NG301−CG321], [CG2R61−CG321−NG301−CG321] and [CG321−CG321−NG301−CG321]. The dihedrals optimized in the **JE22** ligand were [CG205−CG321−CG321−NG301], [CG2R61−CG321−CG321−NG301], [CG2R61−CG321−NG301−CG321], [CG321−CG321−NG301−CG321] and [CG331−CG205−CG321−CG321]. The MCSA dihedral fitting procedure for the dihedrals to the QM PES was done with a linear combination of potentials with periodicities n = 1, 2 and 3. The dihedrals were fitted to reproduce the minima on the PES.

The same equilibration protocol was used for all the simulations, consisting of 10,000 steps of energy minimization, followed by 10 ns of dynamics in the NPT ensemble with timestep equal to 1 fs and 10 ns of dynamics in the NPT ensemble with timestep equal to 2 fs. In the course of the equilibration, restraints on the heavy atoms of the protein were gradually reduced to zero in four stages of 50 ps (1.0, 0.5 0.25 and 0.1 kcal mol for the backbone and 0.5, 0.25 0.125 and 0.05 kcal mol for the sidechain). Restraints on heavy atoms of the ligand were also released in a similar way (1.0, 0.5 0.25 and 0.1 kcal mol run for 50 ps each). Long−range electrostatic interactions were calculated with the Particle Mesh Ewald method using a grid spacing of 1.0 Å [2] and NAMD defaults for spline and κ values. A 12 Å cut−off was applied to non−bonded forces. Both electrostatics and van der Waals forces were smoothly switched off between the switching distance of 10 Å and the cut−off distance of 12 Å, using the default switching function in NAMD. A Verlet neighbour list with pair−list distance of 16 Å was used to evaluate non−bonded neighbouring forces within the pair−list distance [3]. The temperature was controlled at 298 K by coupling to a Langevin thermostat with a damping coefficient of 1 ps^{-1}. A pressure of 1 atm was maintained by coupling the system to a Langevin piston, with a damping constant of 25 ps and a period of 50 ps. [4] The lengths of covalent bonds involving hydrogen atoms were constrained by the Shake algorithm to use a 2−fs time−step [5]. The multi−time step algorithm Verlet−I/r−RESPA62 [6,7] was used to integrate the equations of motion. The software NAMD2.14 [8] was used to perform the molecular dynamics simulations. Each replica was run for 200 ns. In total, 2 μs of dynamics were analyzed after disregarding the equilibration period.

In all the runs, the root mean square deviation (RMSD) of the Cα atoms of the α−helical and β−sheet components of the protein from the original crystallographic structure attained comparable values to the resolution of the crystal and remained almost flat during the rest of the simulations, indicating that CD44 was structurally stable (**Figure S2**). The time evolution of ligand−protein interactions is presented in **Figure S1** for each of the five replicas considered for each of the two systems amounting to 2 μs of dynamics.

It was found that the behaviour of both ligands is similar and stabilized binding poses are observed only in a fraction of the trajectories (**Figure S3**). **JE22** leaves its original binding site at a shallow pocket on the surface of CD44 in all the trajectories: after 25, 40 and over 100 ns of dynamics and almost immediately in the remaining two replicas. The ligand interacts via the benzyl ring with the protein through residue Arg155 and a cluster formed by Glu41, Asn 29 and Thr31, and on the other side with residues Cys81 and Thr80. Subsequently, the contacts with Cys81 and Thr80 are lost and the ligand remains attached to the protein only through the interactions of the benzyl ring. As time progresses, other interactions with nearby residues e.g., Glu41, Asn 29 and Thr31 are established until the ligand finally loses contact with the protein and departs to the solution. In the case of **THIQ−ester**, only in one replica, after the initial departure from the protein surface, the ligand comes back and lands again in a similar position. In all the simulations, random excursions of the ligand to the protein surface are recorded. The atomic interactions are similar to those described for **JE22** in one of the two main poses.

To characterize the influence of the ligand in the structure of the protein, the normalized probability densities of the of the Cα atom RMSD of the protein from the original structure when any atom of either ligand is found at less 3 Å from the protein were computed (see **Figure S4**). It can be observed that the densities are almost identical in the case of the **THIQ−ester** system, implying that the presence or absence of the ligand at the binding pocket is not reflected in changes in the structure of the protein. In the case of the system with the ketone THIQ derivative **JE22**, two populations are characterized but like in the previous case, the overall shape of the distribution is identical with one exception; a higher peak appears when **JE22** is not interacting with the protein; analysis of the trajectories indicates that this difference is not correlated to the presence or absence of the ligand.

THIQ-ester **JE22**

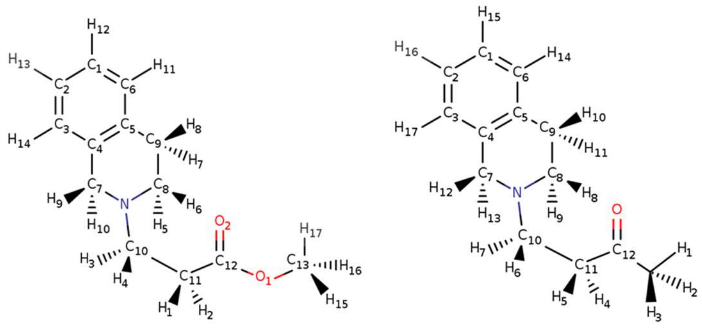

Scheme 1. Chemical structures of the **THIQ−ester** and **JE22** ligands with the nomenclature used in the CHARMM force−field parameterization.

Table S1. CHARMM force field parameterization of **THIQ−ester**. CGenFF 36 atom types and final partial charges.

Name	Type	Partial Charge	Name	Type	Partial Charge
C1	CG2R61	−0.111	N	NG301	−0.378
C2	CG2R61	−0.111	O1	OG302	−0.491

C3	CG2R61	−0.116	O2	OG2D1	−0.626
C4	CG2R61	−0.217	H1/H2	HGA2	0.090
C5	CG2R61	−0.189	H3/H4	HGA2	0.090
C6	CG2R61	−0.113	H10/H9	HGA2	0.090
C7	CG321	0.120	H5/H6	HGA2	0.090
C8	CG321	−0.046	H8/H7	HGA2	0.090
C9	CG321	−0.055	H17/H16/H15	HGA3	0.090
C10	CG321	0.020	H14	HGR61	0.115
C11	CG321	0.348	H13	HGR61	0.115
C12	CG202	0.340	H12	HGR61	0.115
C13	CG331	−0.003	H11	HGR61	0.115

Table S2. CHARMM force field parameterization of **JE22** (**THIQ–ketone**). CGenFF 36 atom types and final partial charges.

**Name**	**Type**	**Partial Charge**	**Name**	**Type**	**Partial Charge**
C1	CG2R61	−0.109	N	NG301	−0.395
C2	CG2R61	−0.123	O	OG302	−0.451
C3	CG2R61	−0.140	H1/H2/H3	HGA2	0.110
C4	CG2R61	0.021	H4/H5	HGA2	0.104
C5	CG2R61	−0.024	H6/H7	HGA2	0.092
C6	CG2R61	−0.122	H8/H9	HGA2	0.096
C7	CG321	−0.051	H10/H11	HGA2	0.099
C8	CG321	−0.038	H12/H13	HGA2	0.096
C9	CG321	−0.192	H14	HGR61	0.117
C10	CG321	−0.065	H15	HGR61	0.110
C11	CG321	−0.123	H16	HGR61	0.114
C12	CG202	0.296	H17	HGR61	0.099
C13	CG331	−0.228			

Table S3. CHARMM force field parameterization of **THIQ–ester**. MP2 equilibrium bond lengths in Å (b_0) and force constants (K_b) in kcal·mol^{-1}·Å^{-2}.

**Bonds**	**Kb (kcal·mol^{-1}·Å^{-2})**	**b_0 (Å)**
CG202 CG321	200.0	1.522
CG202 OG2D1	750.0	1.220
CG202 OG302	150.0	1.334
CG2R61 CG2R61	305.0	1.375
CG2R61 CG321	230.0	1.490
CG2R61 HGR61	340.0	1.080
CG321 CG321	222.5	1.530
CG321 NG301	200.0	1.450
CG321 HGA2	309.0	1.111
CG331 OG302	340.0	1.430
CG331 HGA3	322.0	1.111

Table S4. CHARMM force field parameterization of **JE22** (**THIQ–ketone**). MP2 equilibrium bond lengths in Å (b_0) and force constants (K_b) in kcal·mol^{-1}·Å^{-2}.

**Bonds**	**Kb** (kcal·mol^{-1}·Å^{-2})	**b_0** (Å)
CG205 CG321	330.0	1.500
CG205 CG331	330.0	1.500
CG205 OG2D3	700.0	1.230
CG2R61 CG2R61	305.0	1.375
CG2R61 CG321	230.0	1.490
CG2R61 HGR61	340.0	1.080
CG321 CG321	222.5	1.530
CG321 NG301	200.0	1.450
CG321 HGA2	309.0	1.111
CG331 HGA3	322.0	1.111

Table S5. CHARMM force field parameterization of **THIQ–ester**. CGenFF 36 bond angles in degrees (b_0) and force constants (K_b) in kcal·mol^{-1}·rad^{-2}.

**Angles**	**Kb** (kcal·mol^{-1}·rad^{-2})	**b$_0$** (degrees)
CG321 CG202 OG21	70.0	125.0
CG321 CG202 OG302	55.0	109.0
OG2D1 CG202 OG302	90.0	125.9
CG2R61 CG2R61 CG2R61	40.0	120.0
CG2R61 CG2R61 CG321	45.8	120.0
CG2R61 CG2R61 HGR61	30.0	120.0
CG202 CG321 CG321	52.0	108.0
CG202 CG321 HGA2	33.0	109.5
CG2R61 CG321 CG321	51.8	107.5
CG2R61 CG321 NG301	73.0	109.0
CG2R61 CG321 HGA2	49.3	107.5
CG321 CG321 NG301	57.0	107.0
CG321 CG321 HGA2	26.5	110.1
NG301 CG321 HGA2	32.4	109.0
HGA2 CG321 HGA2	35.5	109.0
OG302 CG331 HGA3	60.0	109.5
HGA3 CG331 HGA3	35.5	108.4
CG321 NG301 CG321	70.0	112.0
CG202 OG302 CG331	40.0	109.6

Table S6. CHARMM force field parameterization of **JE22** (**THIQ–ketone**). CGenFF 36 bond angles in degrees (b_0) and force constants (K_b) in kcal·mol^{-1}·rad^{-2}.

**Angles**	**Kb** (kcal·mol^{-1}·rad^{-2})	**b$_0$** (degrees)
CG321 CG205 CG331	35.0	115.6
CG321 CG205 OG2D3	75.0	122.2
CG331 CG205 OG2D3	75.0	122.2
CG2R61 CG2R61 CG2R61	40.0	120.0
CG2R61 CG2R61 CG321	45.8	120.0
CG2R61 CG2R61 HGR61	30.0	120.0

CG205 CG321 CG321	60.0	113.8
CG205 CG321 HGA2	50.0	109.5
CG2R61 CG321 CG321	51.8	107.5
CG2R61 CG321 NG301	73.0	109.0
CG2R61 CG321 HGA2	49.3	107.5
CG321 CG321 NG301	57.0	107.0
CG321 CG321 HGA2	26.5	110.1
NG301 CG321 HGA2	32.4	109.0
HGA2 CG321 HGA2	35.5	109.0
CG205 CG331 HGA3	50.0	109.5
HGA3 CG331 HGA3	35.5	108.4
CG321 NG301 CG321	70.0	112.0

Table S7. CHARMM force field parameterization of **THIQ–ester**. CGenFF 36 torsion parameters with force constants (K_b) in kcal·mol⁻¹. n is the periodicity.

**Torsions**	**Kb** (kcal·mol^{-1})	**n**	**δ** (degrees)
CG2R61 CG2R61 CG321 CG321	0.230	2	180.0
OG302 CG202 CG321 HGA2	0.000	3	0.0
CG321 CG2R61 CG2R61 CG321	2.400	2	180.0
HGR61 CG2R61 CG2R61 HGR61	2.400	2	180.0
NG301 CG321 CG321 HGA2	0.160	3	0.0
HGA2 CG321 CG321 HGA2	0.220	3	0.0
CG2R61 CG2R61 CG321 HGA2	0.002	6	0.0
OG2D1 CG202 CG321 HGA2	0.000	6	180.0
CG2R61 CG2R61 CG2R61 CG2R61	3.10	2	180.0
CG2R61 CG2R61 CG2R61 CG321	3.10	2	180.0
CG321 CG2R61 CG2R61 HGR61	2.40	2	180.0
HGA2 CG321 NG301 CG321	0.10	3	0.0
CG2R61 CG2R61 CG321 NG301	1.00	2	180.0
HGA3 CG331 OG302 CG202	0.00	3	0.0
CG2R61 CG321 CG321 HGA2	0.04	3	0.0
CG202 CG321 CG321 HGA2	0.195	3	0.0

CG2R61 CG2R61 CG2R61 HGR61	4.20	2	180.0
OG302 CG202 CG321 CG321	0.166	2	180.0
CG321 CG321 NG301 CG321	0.4190	2	0.0
CG321 CG321 NG301 CG321	0.914	3	0.0
CG321 CG202 OG302 CG331	2.528	2	180.0
OG21 CG202 OG302 CG331	1.602	1	180.0
OG21 CG202 OG302 CG331	1.922	2	180.0
OG2D1 CG202 CG321 CG321	0.492	6	0.0
CG2R61 CG321 NG301 CG321	1.094	1	0.0
CG2R61 CG321 NG301 CG321	2.292	2	0.0
CG2R61 CG321 CG321 CG321	0.344	3	0.0
CG2R61 CG321 CG321 NG301	0.059	3	0.0
CG202 CG321 CG321 NG301	2.366	3	180.0
CG202 CG321 CG321 NG301	0.411	3	0.0

Table S8. CHARMM force field parameterization of **JE22** (**THIQ–ketone**). CGenFF 36 torsion parameters with force constants (K_b) in kcal·mol⁻¹. n is the periodicity.

**Torsions**	**Kb** (kcal·mol^{-1})	**n**	**δ** (degrees)
OG2D3 CG205 CG321 HGA2	0.000	3	0.0
HGR61 CG2R61 CG2R61 HGR61	2.400	2	180.0
NG301 CG321 CG321 HGA2	0.16	3	0.0
OG2D3 CG205 CG331 HGA3	0.000	3	0.0
HGA2 CG321 CG321 HGA2	0.220	3	0.0
CG2R61 CG2R61 CG321 HGA2	0.002	6	0.0
CG2R61 CG2R61 CG2R61 CG2R61	3.10	2	180.0
OG2D3 CG205 CG321 CG321	0.75	1	180.0
OG2D3 CG205 CG321 CG321	0.18	2	180.0
OG2D3 CG205 CG321 CG321	0.065	3	180.0
OG2D3 CG205 CG321 CG321	0.030	6	0.0
CG2R61 CG2R61 CG2R61 CG321	3.10	2	180.0
CG205 CG321 CG321 HGA2	0.195	3	0.0
CG321 CG2R61 CG2R61 HGR61	2.40	2	180.0

HGA2 CG321 NG301 CG321	0.10	3	0.0
CG331 CG205 CG321 HGA2	0.10	3	0.0
CG321 CG205 CG331 HGA3	0.10	3	0.0
CG2R61 CG321 CG321 HGA2	0.04	3	0.0
CG202 CG321 CG321 HGA2	0.195	3	0.0
CG2R61 CG2R61 CG2R61 HGR61	4.20	2	180.0
CG331 CG205 CG321 CG321	2.179	1	0.0
CG331 CG205 CG321 CG321	0.193	2	180.0
CG331 CG205 CG321 CG321	0.252	3	0.0
CG331 CG205 CG321 CG321	0.096	6	180.0
CG331 CG205 CG321 CG321	0.332	1	180.0
CG331 CG205 CG321 CG321	0.843	2	0.0
CG331 CG205 CG321 CG321	0.085	3	180.0
CG331 CG205 CG321 CG321	0.428	6	0.0
CG321 CG321 NG301 CG321	1.991	1	180.0
CG321 CG321 NG301 CG321	0.048	3	0.0
CG321 CG321 NG301 CG321	0.660	1	0.0
CG321 CG321 NG301 CG321	2.200	2	0.0
CG321 CG321 NG301 CG321	1.121	3	0.0
CG205 CG321 CG321 NG301	1.375	3	180.0
CG321 CG2R61 CG2R61 CG321	0.037	2	180.0
CG2R61 CG321 NG301 CG321	0.002	2	180.0
CG205 CG321 CG321 NG301	0.362	3	180.0
CG2R61 CG321 NG301 CG321	0.503	1	180.0
CG2R61 G321 NG301 CG321	0.86	2	180.0
CG2R61 CG321 NG301 CG321	0.338	3	0.0
CG2R61 CG321 CG321 NG301	0.288	3	180.0
CG2R61 CG2R61 CG321 CG321	0.018	2	180.0
CG2R61 CG2R61 CG321 CG321	0.829	2	0.0
CG2R61 CG2R61 CG321 N301	0.150	2	0.0

CG2R61 CG2R61 CG321 NG301	0.938	2	0.0

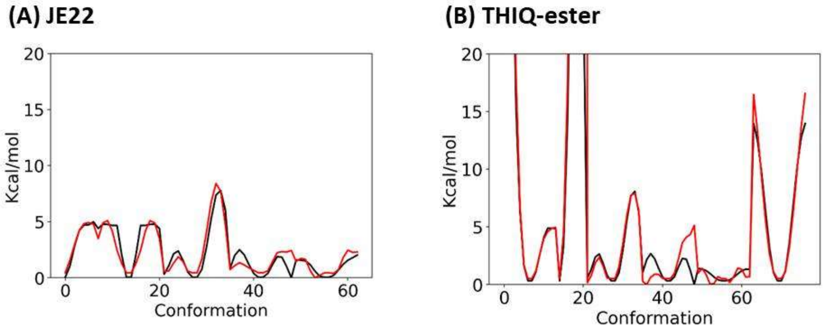

Figure S1. Comparison of the potential energy surface (PES) from quantum mechanics calculations (QM) in black and the fitted torsions in red for some the torsional angles of (**A**) **JE22** and (**B**) **THIQ–ester** optimized.

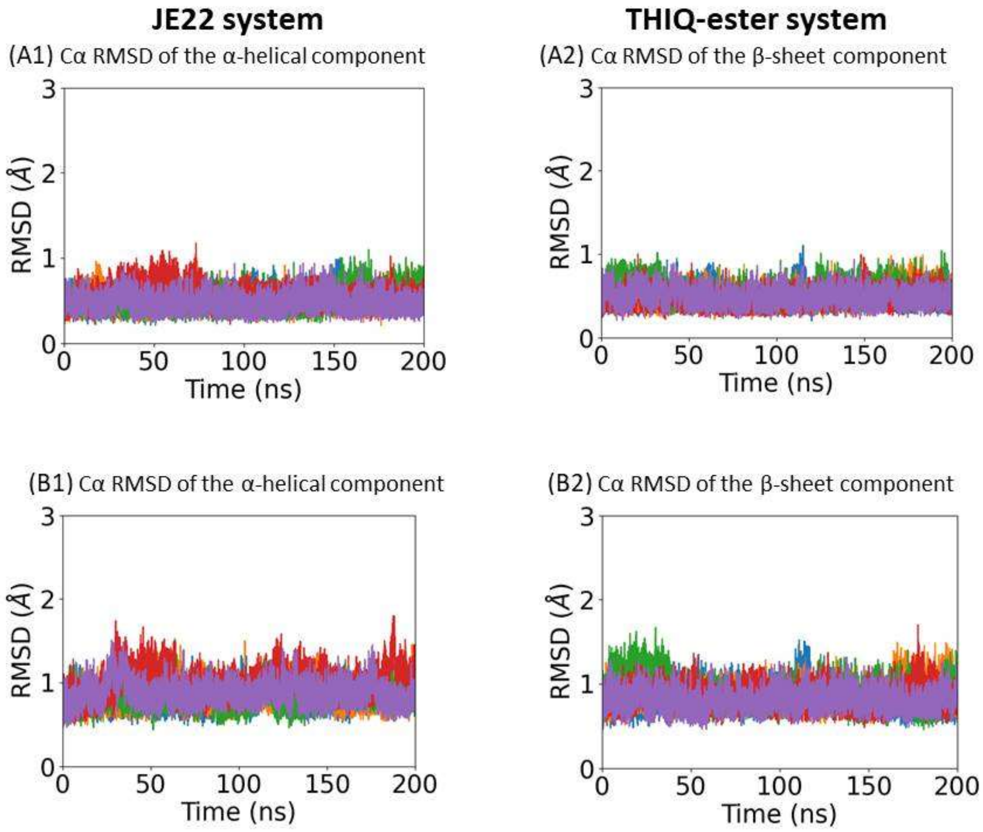

Figure S2. Evolution of the Cα root–mean–square (RMSD) deviation of different structural elements of CD44 from the initial structure as a function of time for the system with (**A**) **JE22** or (**B**) **THIQ–ester**. Contributions from the α–helical (A1 & B1) and β–sheet (A2 & B2) components of the protein in each replica are shown in different colour.

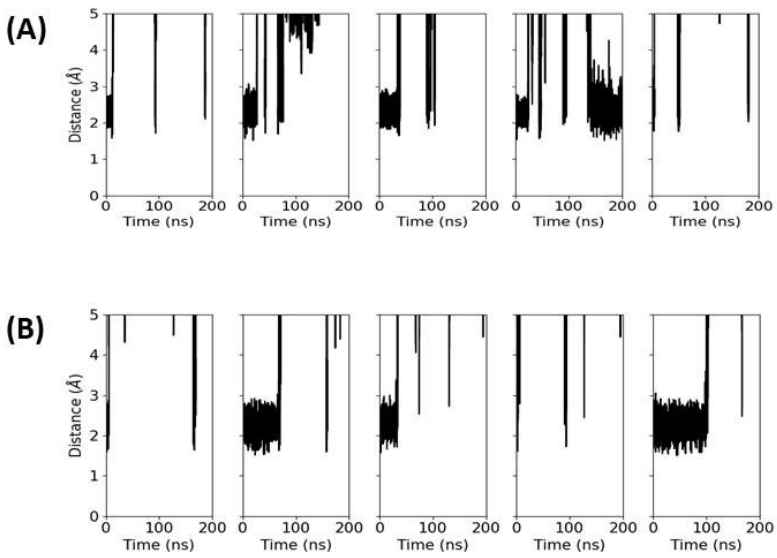

Figure S3. Evolution in time of the minimum distance between any atom of the ligand, either (**A**) **JE22** or (**B**) **THIQ–ester**, and any atom of the binding site composed by N29 T31 E41 R80 C81 R82 R155, for each of the five replicas.

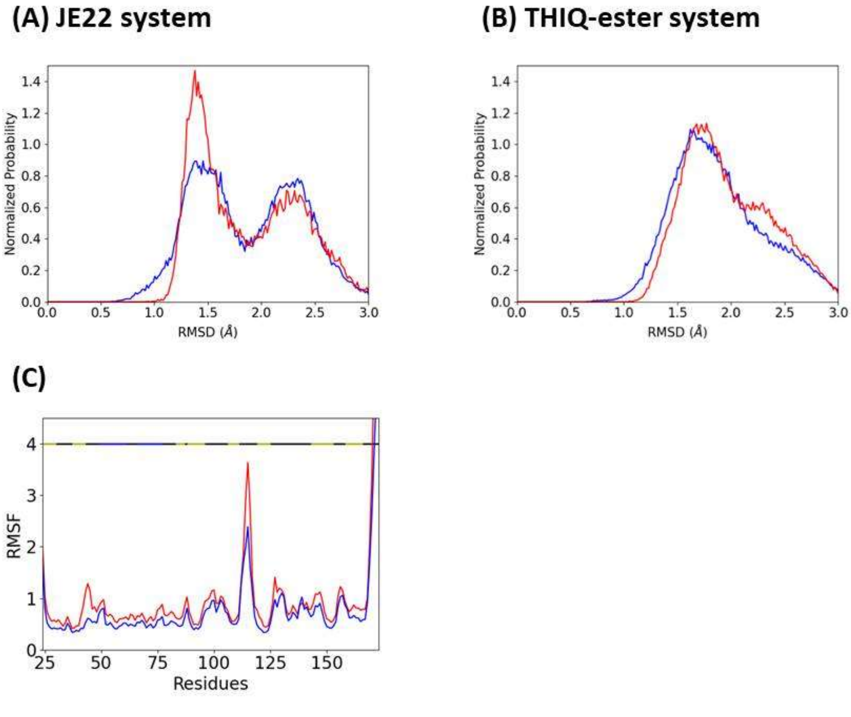

Figure S4. (**A** and **B**) Normalized probability density of the Cα RMSD of the protein with respect to the first frame of the simulation, when any atom of either ligand is at less than 3 Å (blue) from the protein or when the ligand is further than 3 Å from the protein (red). (**C**) RMSF values of the protein using as a reference the starting frame of the simulation in the two representative situations described in (**A**). The different structural elements are indicated in the horizontal bar above the RMSF data; helices are in blue, loops, coils and turns in black, and βsheet in yellow. The peak in red in (**A**) does not correlate with the presence or absence of the ligand in the binding site; it is a result of the movement of some loop.

2. Synthesis and characterization of 4–(3,4–dihydroisoquinolin–2(1H)–yl)butan–2–one (JE22)

To a solution of methyl vinyl ketone (0.66 mL, 7.88 mmol) and cupper bromide (I) (0.011 g, 0.078 mmol) in CH_2Cl_2 (25 mL), THIQ (1 mL, 7.88 mmol) was added dropwise at 0 °C. The reaction mixture was stirred for 30 min at 0 °C and then, 64 h at room

temperature. It was concentrated and the residue was purified by flash chromatography using a gradient of EtOAc/hexane as eluent (1:3 → 1:1) to yield a light−yellow oil (1.211 g, 76%).

^{1}H NMR (400 MHz, $CDCl_3$) δ 7.16 – 7.06 (m, 3H), 7.01 (d, *J* = 5.8 Hz, 1H), 3.69 (s, 2H), 2.96 – 2.85 (m, 4H), 2.84 – 2.76 (m, 4H), 2.20 (s, 3H). ^{13}C NMR (101 MHz, $CDCl_3$) δ 207.61, 133.83, 133.73, 128.77, 126.68, 126.53, 125.93, 55.80, 52.33, 50.89, 41.41, 30.32, 28.67. HRMS (ESI−TOF) (m/z) calcd. for $C_{13}H_{18}NO$ $(M + H)^+$ 204.1310, found 204.1392.

$^{1.}$ H and ^{13}C NMR spectra of ***JE22***

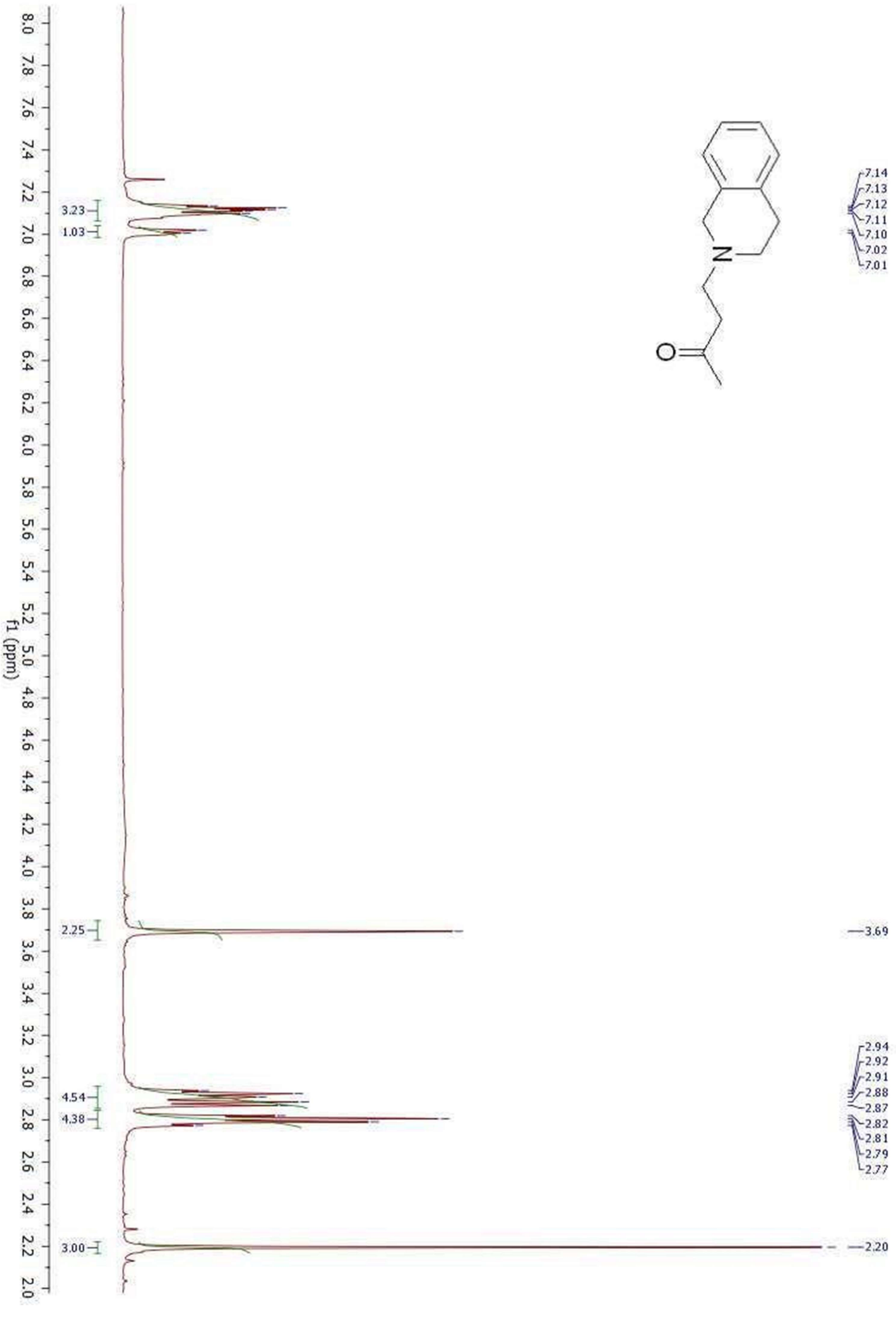

Figure S5. ^{1}H NMR **JE22**.

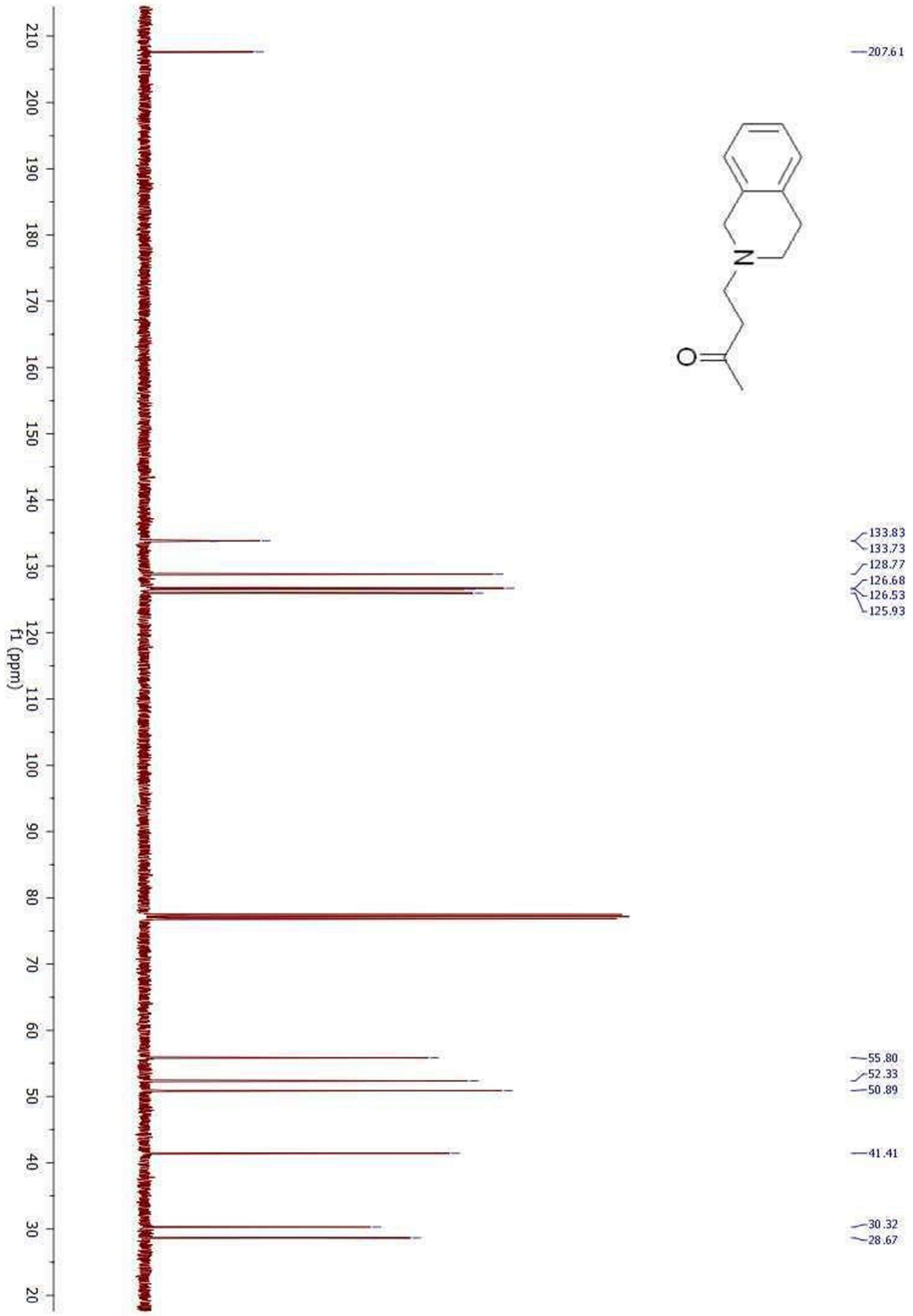

Figure S6. ¹³C NMR **JE22**.

3. General experimental methods

3.1. Synthesis of ***Naked–NPs*** *(**1**)*

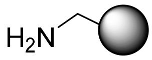

1

PVP (Mw 29,000, 0.05 g, 1.7 μmol, Sigma−Aldrich) was dissolved in 92% ethanol/8% water for a final volume of 10 mL, and deoxygenated via argon bubbling. AIBN (7 mg, 42.4 μmol) was dissolved in styrene (freshly washed, 0.5 mL) with VBAH (7 mg, 41.3 μmol) and DVB (freshly washed, 4.65 μL) [9]. The dispersion was deoxygenated with argon bubbling before addition to the PVP/Ethanol solution.

The mixture was stirred under argon for 1 hour before heating to 68 °C for 15 h. NPs were obtained by centrifugation (11,000 G, 15 min) and washed with methanol (2 × 10 mL) and water (2 × 10 mL). Finally, NPs were stored in water (10 mL) at 4°C.

Particle size distribution: mean diameter: 382.5 nm, PDI: 0.13.

Loading (Ninhydrin): 0.064 mmol / g.

Number of particles per gram: 1.42×10^{13}.

3.1.1. Characterization of **Naked−NPs** (**1**)

3.1.1.1. Solid content (SC) of the emulsion (%)

A known mass of a suspension of polystyrene NPs (0.5−1 mg, suspended in water) was placed in a watch glass, covered with aluminium foil, dried at 25 °C for 15 h, weighed and reweighed to give the mass of NPs. The solid content was then calculated according to the following equation:

$$\%\,SC = \frac{M}{Vs} \times 100 \quad (1)$$

where M = mass of NPs (mg), Vs = Volume of suspension (μL).

SC: 3%, 3 mg of NPs in 100 μL of solution.

3.1.1.2. Calculation of number of particles per gram

$$N = \frac{6 \times 10^{12}}{\pi \times \rho \times d^3} \quad (2)$$

where N = Number of particles/g for dry powder, ϱ= Density of solid spheres (g/cm^3), which is 1 g/cm^3 for polystyrene, d= Mean diameter (nm).

Result: N = 1.42×10^{13} NPs per gram.

3.1.1.3. Calculation of loading of NPs using Fmoc NPs test

Fmoc−(x)−NPs (where x is Fmoc−PEG−OH) were resuspended in 1 mL of 20% piperidine in DMF (3 × 20 min) after which the beads were washed by centrifugation three times, the supernatants combined and the loading was calculated according to the following equation:

$$Loading\left(\frac{mmol}{g}\right) = \frac{(A_{302} \times V)}{(\varepsilon_{302} \times d \times W)} \times 1000 \quad (3)$$

where A_{302} : Absorbance measured at 302 nm, V_{mL}: Volume of combined supernatants, ε_{302}: Molar Extinction Coefficient (7800 $M^{-1}cm^{-1}$) and W_{mg}: Mass of beads.

Result: Loading (Fmoc test): 0.064 mmol/g

3.1.1.4. Qualitative ninhydrin test

The reaction control was determined by qualitative ninhydrin test. 12 μL samples of NPs in methanol (3% SC) in a 0.5 mL capacity eppendorf were washed with methanol and centrifuged after which 6 μL of reagent A and 2 μL of reagent B were added. Mix well and heat to 100 °C for 3 min. Blue stained resin beads indicate the presence of primary amines.

3.2. Determination of NPs concentration (NPs/μL) by spectrophotometric method

NPs concentration (NPs per microliter) was determined by a spectrophotometric method as described previously [10]. Briefly, measurement of turbidity optical density at 600 nm of polystyrene NP suspensions was performed, based on nephelometric principals. Light going through NP suspensions is scattered via reflection, refraction and diffraction phenomena and the intensity of the scattered light, which are proportional to number of NPs in suspension, is recorded by standard spectrophotometers. In this way, calibrate standard curves were obtained for amino–methyl cross–linked polystyrene NPs of 385 nm by NP known concentrations. Calibration curves fitted linear regression models by which the number of NPs per microliter corresponding to one unit of OD600 for each size could be determined. Thus, these curves using initial batches of NP suspensions permitted us to estimate the number of NPs in final batches, which underwent multiple handling procedures, by OD600 measurement of 1 μL (**Figure S7**).

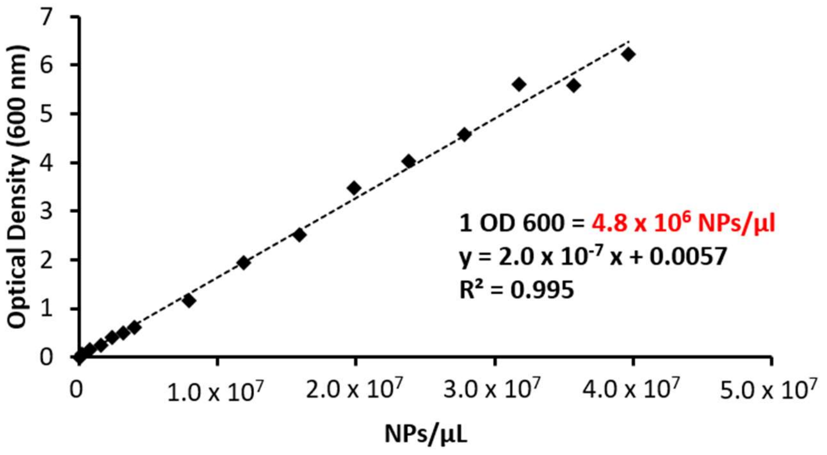

Figure S7. Calibration standard curve of concentration of nanoparticles (OD 600).

4. Summarized table of the synthesized nanoparticles

Table S9. Code, name and structure of synthesized nanoparticles.

Code	Name	Structure
1	Naked–NPs	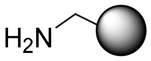
2	PEGylated–NPs	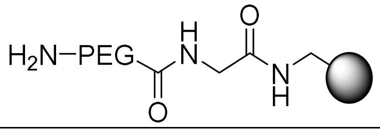
3	COOH–NPs	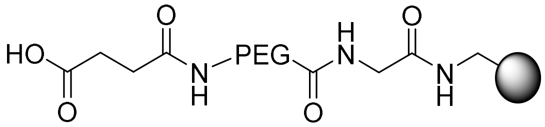
4	Hydrazine–NPs	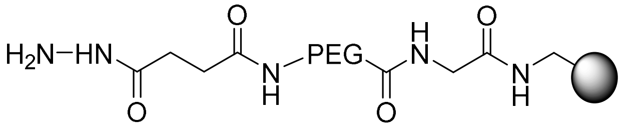
5	JE22–NPs	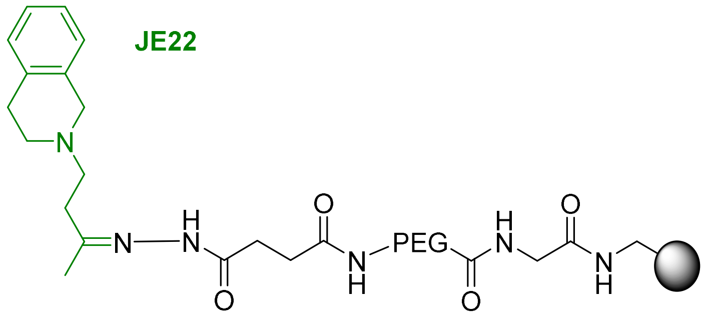

5. Characterization of JE22–NPs (5) before and after incubation in pH= 5 for 5 days by DLS

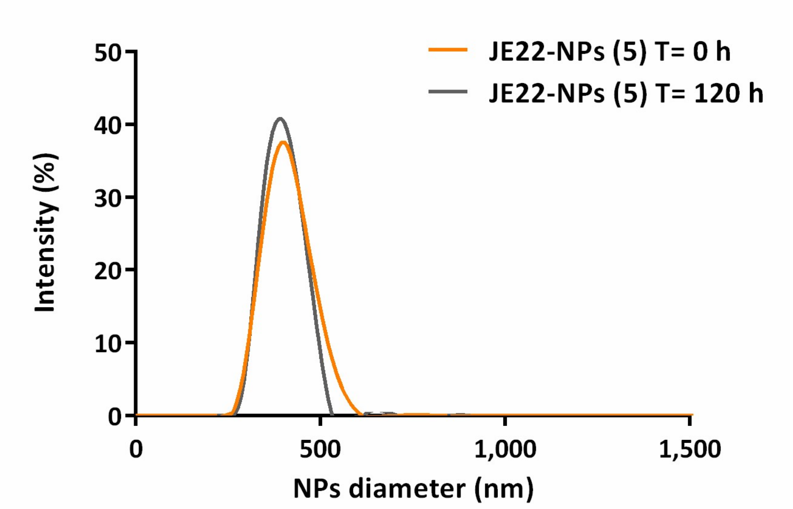

Figure S8. DLS analysis of **JE22–NPs** (**5**) before and after incubation in PBS pH= 5 for 5 days.

6. Stability study of JE22–NPs (5) by zeta potential analysis

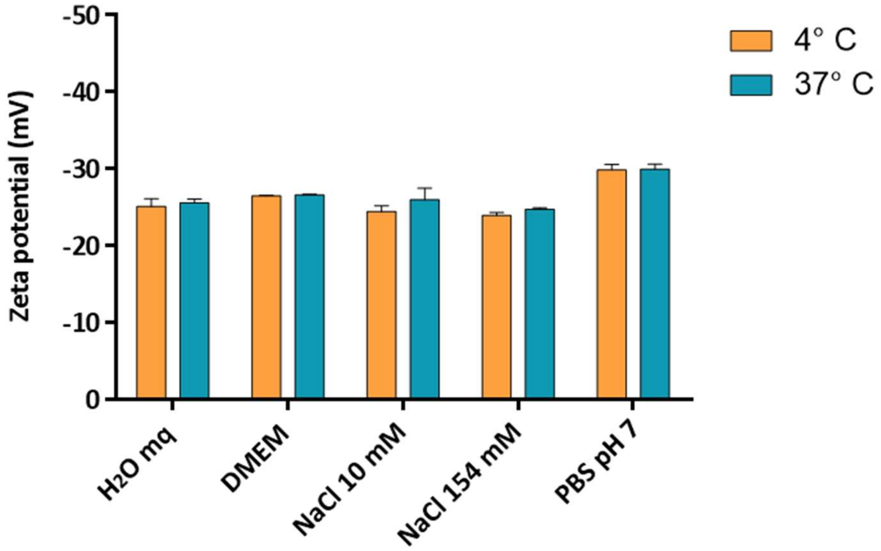

Figure S9. Bar graph showing the zeta potential (mV) values of **JE22–NPs** (**5**) after incubation for 24 h at 4 °C and 37 °C in sterile ultrapure H2O mq, DMEM, NaCl 10 mM, NaCl 154 mM and PBS pH = 7. Data represent the mean ± SD of three independent experiments.

7. Absorbance and calibration standard curves of JE22 of JE22−NPs (5)

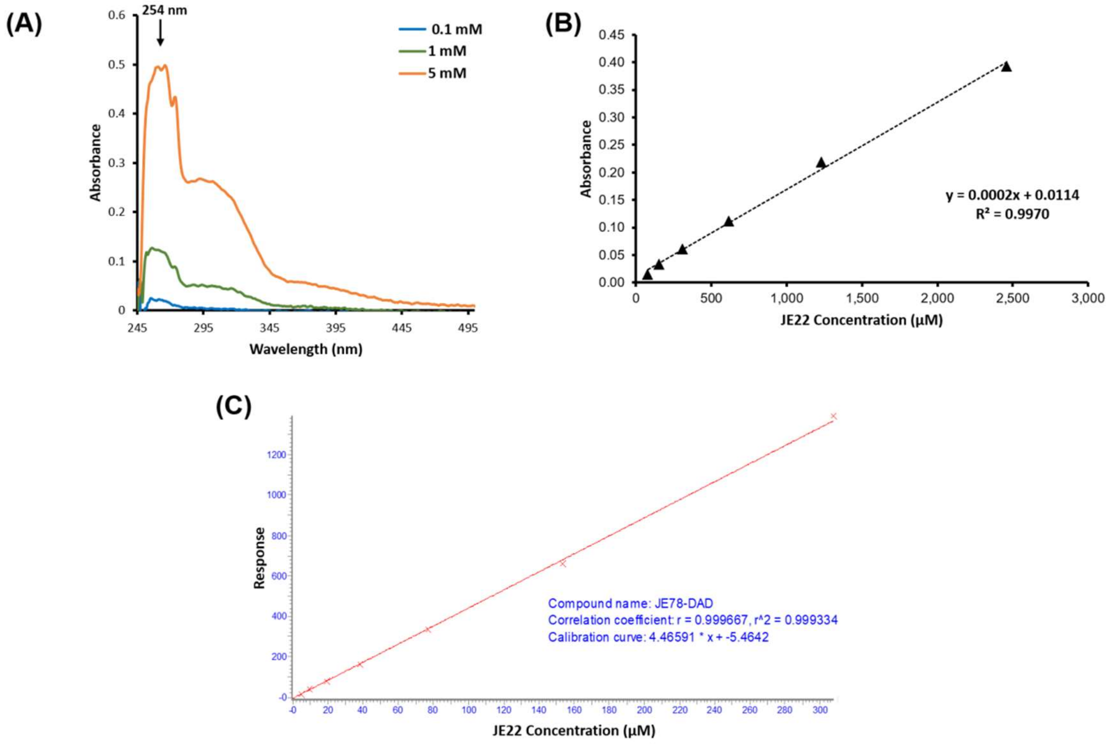

Figure S10. (**A**) Spectrophotometry determination of selected wavelength of **JE22**. (**B**) Calibration standard curve of **JE22** obtained by spectrophotometry. (**C**) Calibration curve of **JE22** acquired by HPLC.

8. Fluorescent labelling of JE22−NPs (5)

JE22−NPs (**5**) were treated with a solution of Sulfo−Cy5−NHS (1 eq.) in anhydrous DMF (1 mL) and DIPEA (1 eq.) was added then the suspension was mixed on the Thermomixer at 1400 rpm for 15 h at 25 °C [11]. Finally, NPs were obtained by centrifugation and subsequently washed several times with DMF (3 × 1 mL), MeOH (3 × 1 mL) and sterile ultrapure water (3 × 1 mL).

9. Flow cytometry analysis of CD44 expression in MDA−MB−231 and MCF−7 cells

Adherent MDA−MB−231 and MCF−7 cells were trypsinized and counted in order to have 8×10^5 cells/eppendorf tube. Cells were centrifuged, supernatant removed and pellets resuspended in a blocking buffer (98 μL). Anti−CD44−FITC antibody (Miltenyi Biotec) was added (2 μL) and cells incubated in dark and ice for 10 minutes. Cells were centrifuged, pellets resuspended in PBS and samples analyzed by flow cytometry using FACSCanto II (Becton Dickinson & Co.) and Flowjo® 10 software (**Figure S11**).

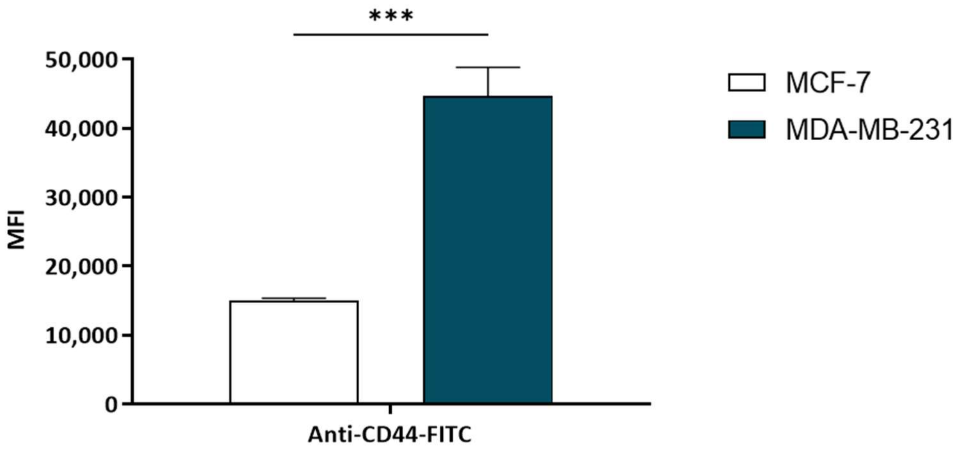

Figure S11. Flow cytometry analysis of the expression of CD44 in MDA−MB−231 and MCF−7 cells. Representative bar graph comparing the mean fluorescence intensity (MFI) of cancer cell lines after incubation with anti−CD44−FITC. Error bars: ± SD from n = 3; ***$p < 0.001$ (ANOVA).

10. Effect of JE22−NPs (5) on cell viability in MCF−7 cell line

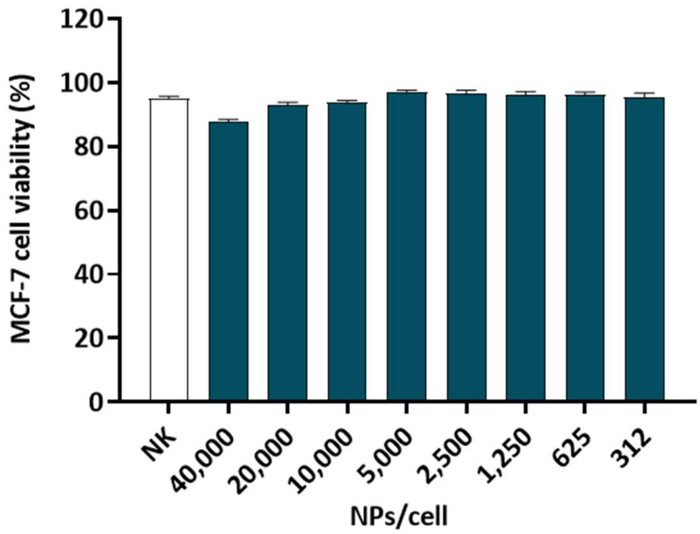

Figure S12. Bar graph showing the effect of **JE22−NPs** (**5**) on cell viability of MCF−7 cell line. A range of nanoparticles from 312 to 40,000 was tested. **Naked−NPs** (**1**) **(NK)** were used as control. Data represent the mean ± SD of three independent experiments.

11. Competitive assay of JE22–NPs (5) in MDA–MB–231 cell viability

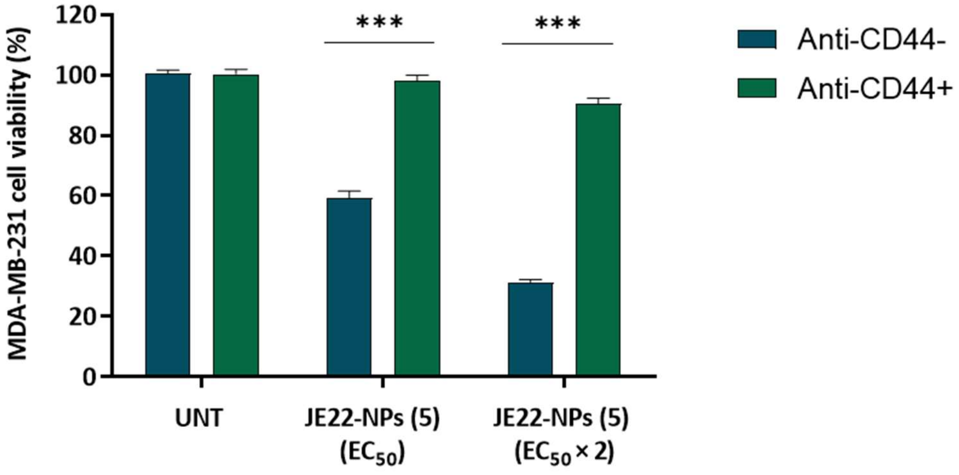

Figure S13. MDA–MB–231 cell viability after treatment with **JE22–NPs** (**5**) (EC_{50} and EC_{50} × 2) pre-incubated with anti–CD44 antibody (Anti–CD44+, 2.5 μM, 40 min). Untreated cells (UNT) and cells treated with **JE22–NPs** (**5**) without preincubation with anti–CD44 antibody (Anti–CD44–) were used as controls. Error bars: ± SD from n = 3; ***$p < 0.001$ (ANOVA).

12. Dose–response curve of MDA–MB–231 treated with JE22–NPs (5), represented in NPs/cell

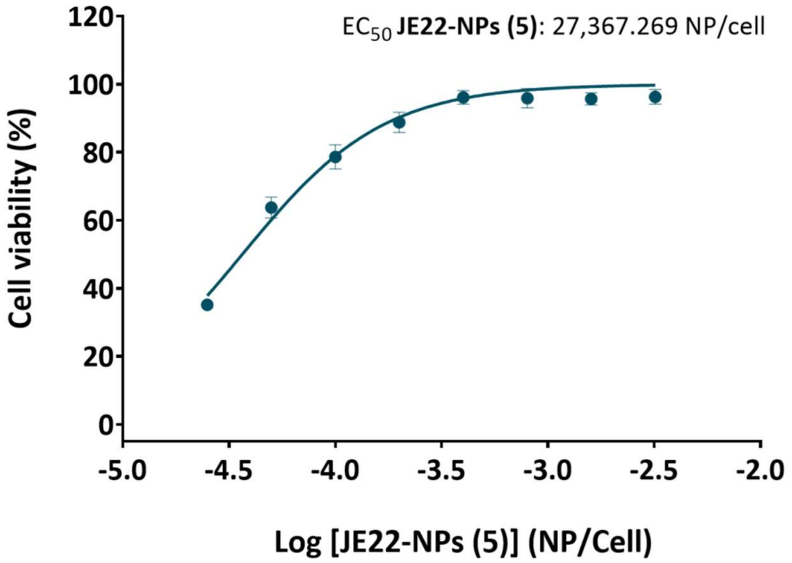

Figure S14. Dose–response curve (percentage of cell viability *versus* concentration) of MDA–MB–231 after treatment with **JE22–NPs** (**5**), expressed in NPs/cell. EC_{50} value was obtained by the logarithm *versus* normalized response: variable slope using the GraphPad software.

13. Study of pH effect on MDA–MB–231 cell viability

MDA–MB–231 cells were seeded in a 96–well plate format (1,000 cells/well) and incubated for 24 h. Each well was then replaced with DMEM media at pH= 5 and incubated for 1.5, 3 and 6 h. Media was replaced with pH=7.4 DMEM media and cell viability tested at day 5 using PrestoBlue™ cell viability reagent. Cells incubated with normal DMEM media were used as control. Each condition was performed in triplicates (**Figure S15**).

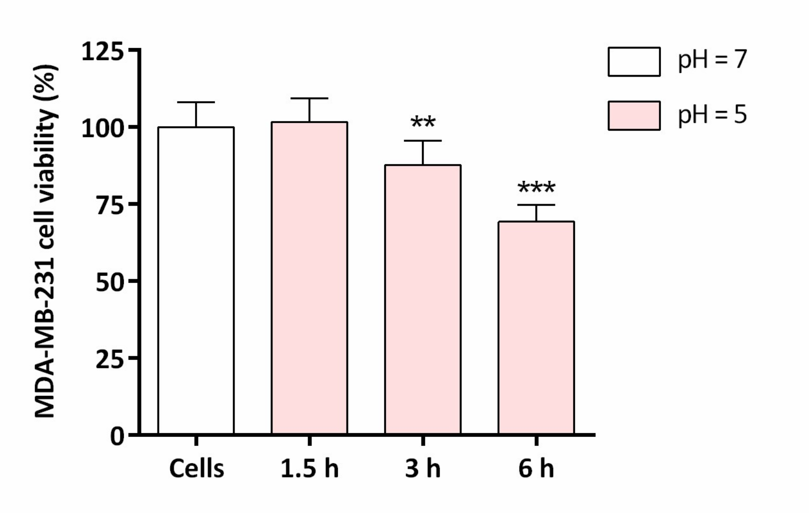

Figure S15. Viability of MDA–MB–231 cells incubated for 1.5, 3 and 6 h with DMEM media at pH= 5. Error bars: ± SD from n = 3; ***p* < 0.01, ****p* < 0.001 (ANOVA).

14. Cytotoxic analysis of JE22–NPs (5) in non–cancerous HEK–293 cell line

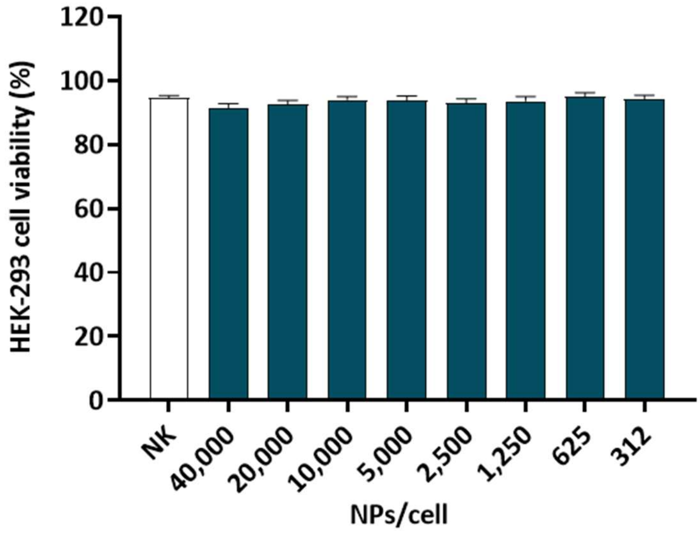

Figure S16. Bar graph showing the effect of **JE22–NPs** (**5**) on cell viability of HEK–293 cell line. A range of nanoparticles from 312 to 40,000 was tested. **Naked–NPs** (**1**) (NK) were used as control. Data represent the mean ± SD of three independent experiments.

15. Confocal microscopy analysis of JE22−NPs (5) with CD44+cells

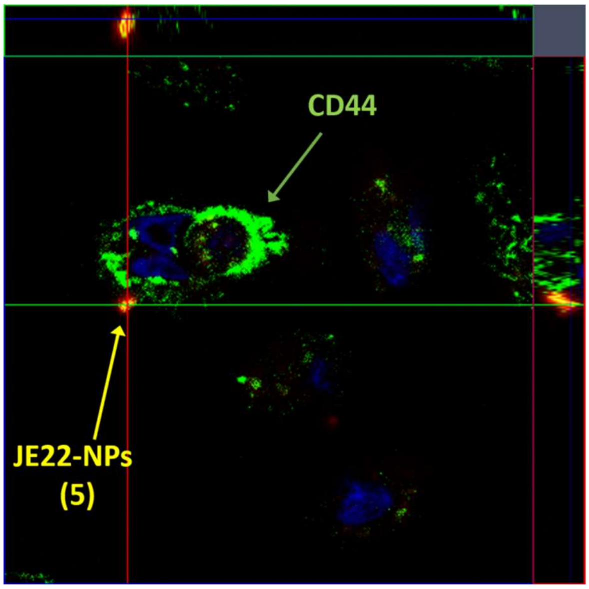

Figure S17. Confocal microscopy orthogonal (xy, xz and yz) view representing the planes of intersection at the position of green cross line. The maximum intensity projection of the z−stack is shown for blue nuclei (DAPI), green anti−human CD44 (FITC) and red for **JE22−NPs** (**5**) (APC).

16. Effect of JE22−NPs (5) on apoptosis in MCF−7 cell line

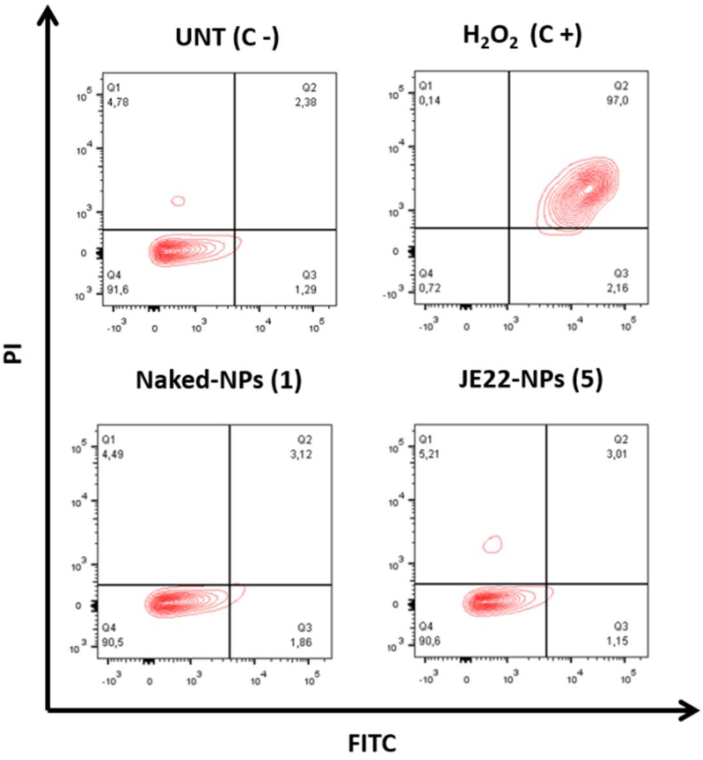

Figure S18. Effect of **JE22−NPs** (**5**) in MCF−7 cell apoptosis. Representative scatter plots of PI (y−axis) versus FITC (x−axis) of cells after treatment with **JE22−NPs** (**5**) (20,000 NPs/cell) and **Naked−NPs** (**1**) (20,000 NPs/cell) for 24 h. The cells were analyzed after double staining with Annexin V−FITC/PI by flow cytometry. Dot plots of H_2O_2 (2 mM, treatment of 4 h) and untreated cells were represented as positive and negative control, respectively. Q1−4 quadrants represent necrotic (Annexin V−FITC−, PI+), late (Annexin V−FITC+, PI+) and early (Annexin V−FITC+, PI−) apoptotic and viable (Annexin V−FITC−, PI−) cells, respectively.

17. References

1. Kim, S.; Lee, J.; Jo, S.; Brooks III, C.L.; Lee, H.S.; Im, W. CHARMM−GUI ligand reader and modeler for CHARMM force field generation of small molecules. *J. Comput. Chem.* **2017**, *38*, 1879–1886.
2. Essmann, U.; Perera, L., Berkowitz, M.L.; Darden, T.; Lee, H.; Pedersen, L.G. A smooth particle mesh Ewald method. *J. Chem. Phys*. **1995**, *103*, 8577–8593.
3. Verlet, L. Computer "Experiments" on Classical Fluids. I. Thermodynamical Properties of Lennard−Jones Molecules. *Phys. Rev*. **1967**, *159*, 98–103.
4. Feller, S.E., Zhang, Y., Pastor, R.W.; Brooks, B.R. Constant pressure molecular dynamics simulation: The Langevin piston method. *J. Chem. Phys*. **1995**, *103*, 4613–4621.
5. Miyamoto, S.; Kollman, P.A. SETTLE−an analytical version of the Shake and Rattle algorithm for rigid water models. *J. Comput. Chem.* **1992**, *13*, 952–962.
6. Tuckerman, M.; Berne, B.J.; Martyna, G.J. Reversible Multiple Time Scale Molecular−Dynamics. *J. Chem. Phys.* **1992**, *97*, 1990–2001.
7. Phillips, J.C.; Braun, R.; Wang, W.; Gumbart, J.; Tajkhorshid, E.; Villa, E.; Chipot, C.; Skeel, R.D.; Kale, L.; Schulten, K. Scalable molecular dynamics with NAMD. *J. Comput. Chem.* **2005**, *26*, 1781–1802.
8. Phillips, J.C.; Hardy, D.J.; Maia, J.D.C.; Stone, J.E.; Ribeiro, J.V.; Bernardi, R.C.; Buch, R.; Fiorin, G.; Henin, J.; Jiang, W.; McGreevy, R.; Melo, M.C.R.; Radak, B.K.; Skeel, R.D.; Singharoy, A.; Wang, Y.; Roux, B.; Aksimentiev, A.; Luthey−Schulten, Z.; Kale, L.V.; Schulten, K.; Chipot, C.; Tajkhorshid, E. Scalable molecular dynamics on CPU and GPU architectures with NAMD. *J. Chem. Phys*. **2020**, *153* (4).
9. Unciti−Broceta, A.; Johansson, E.; Yusop, Rahimi M.; Sánchez−Martín, R.; Bradley, M. Synthesis of polystyrene microspheres and functionalization with Pd0 nanoparticles to perform bioorthogonal organometallic chemistry in living cells. *Nat. Protoc.* **2012**, *7*, 1207–1218.
10. Unciti−Broceta, J.; Cano−Cortés, V.; Altea−Manzano, P.; Pernagallo, S.; Díaz−Mochón, J.; Sánchez−Martín, R. Number of Nanoparticles per Cell through a Spectrophotometric Method−A key parameter to Assess Nanoparticle−based Cellular Assays. *Sci. Rep*. **2015**, *5* (1), doi:10.1038/srep10091.
11. Alexander, L.; Sánchez−Martín, R.; Bradley, M. Knocking (Anti)−Sense into Cells: The Microsphere Approach to Gene Silencing. *Bioconjug. Chem*. **2009**, *20*, 422–426.
